# Effect of Technological Process on True Retention Rate of Eicosapentaenoic and Docosahexaenoic Acids, Lipid Oxidation and Physical Properties of Canned Smoked Sprat (*Sprattus sprattus*)

**DOI:** 10.1155/2021/5539376

**Published:** 2021-06-01

**Authors:** Zdzisław Domiszewski, Sylwia Mierzejewska

**Affiliations:** Department of Food Industry Processes and Facilities, Koszalin University of Technology, Koszalin 75-620, Poland

## Abstract

In connection with the severe deficiencies of EPA and DHA in the human diet, the industry should provide inexpensive fish products that are characterized by the appropriate lipid quality. The influence of the technological process on true retention rate of EPA and DHA, indicators of lipid oxidation and physical properties, of canned smoked sprat in oil was investigated. It was assumed that the double dose of heat during the technological process (smoking/sterilization) can significantly affect the quality of lipids. The study was carried out on fresh fish and after frozen storage. After smoking, the percentage of EPA and DHA in lipids did not change significantly, while the content of these acids per wet weight (g/100 g) increased by about 20%. During smoking, a faster increase in oxidation products was observed in frozen fish (increase by 22%-36%) than in fresh fish (increase by 31%-54%). Sterilization caused EPA and DHA to be “regrouped” from the fish to the oil rather than their physical losses. After sterilization, the fish retained 70%-77% EPA and DHA content (the rest passed into the oil). EPA and DHA losses were 8.5% higher in canned products obtained from frozen fish compared to fresh fish. True retention should be used to assess changes in EPA and DHA content in fish after sterilization (and not the expression of EPA and DHA content in % or g/100 g). A better indicator of changes in the physical parameters of canned fish after sterilization is the analysis of the proportion of the water layer rather than mass measurement. Despite the double dose of heat that occurred during the canned sprat production process, the peroxide value in fish and in oil did not exceed 10 (mEqO_2_/kg of lipid) and p-anisidine value did not exceed 20. This means that these lipids were characterized by good quality.

## 1. Introduction

One of the most valuable compounds found in fish are two long-chain fatty acids (LCF): eicosapentaenoic acid (EPA) and docosahexaenoic acid (DHA). These compounds belong to the polyenoic fatty acids of the n-3 family (n-3 PUFA). These acids are known, for example, for their antiallergenic [1] and anticancer properties [2]. Although consumer awareness concerning fish and polyunsaturated fatty acid n-3 PUFA is subjected to an increase, due to relatively high prices, fish consumption in many countries is low. It is estimated that even over 80% of people do not consume the lowest recommended daily dose of EPA and DHA [3]. Therefore, preference should be given to producing fish products that are safe, cheap, and contain high levels of EPA and DHA.

A characteristic feature of canned food obtained from oily fish (in addition to safety and long shelf life) is the low price of EPA and DHA (converted to daily requirements) [4]. One of the fish species which is characterized by nonthreatened catches, low price, and high PUFA content is Baltic sprats. Typical products obtained from sprat include canned and smoked fish.

The available scientific articles lack information on the effects of smoking and sterilization on fish lipid quality. The scientific articles concerned either smoked fish or canned fish. The results of fatty acid (FA) composition in canned fish have so far been mostly presented in percentage form. A review of the literature shows that in order to estimate the actual n-3 LC PUFA content in food, the results of analyses should be given in absolute (e.g., g/100 g) rather than relative (e.g., %) units [5, 6]. The different units in which the fatty acid profile, cooking loss, and oil absorption by meat can be expressed can make it difficult to track the changes in EPA and DHA in canned fish. The complexity of this issue is demonstrated in the study carried out by Cheung et al. [7]. These authors found an increase in DHA content in fish after grilling and shallow frying. However, after taking into account true retention rates, it turned out that between 58% and 83% of the original DHA content in relation to raw fish was left after heat treatment.

An important problem in the industrial production of canned fish is the change of physical parameters of fish after sterilization (weight loss). This directly affects, not only production efficiency [8] but also the nutritional value of the product [9].

The aim of this study was to investigate the influence of technological processes (under industrial conditions) on true retention of EPA and DHA and physical properties of canned smoked sprat. It was assumed that the double dose of heat that occurs during the technological process (smoking/sterilization) can significantly affect EPA and DHA content. The study mainly focused on these two acids as they are considered to be very thermolabile. So far, research on canned food has not taken into account the loss of fish weight after sterilization and EPA and DHA content in whole canned food (in solid parts : fish and liquid : oil). It is only known from the literature that EPA and DHA are present in the liquid parts of canned food [10, 11]; however, it is not known how much of them “passed” there from the fish part.

## 2. Materials and Methods

### 2.1. Materials

The study was carried out on fresh sprats (2 batches) and after frozen storage (2 batches). Mean sprat weight was 10.1 g ± 1.6 g and length 11.2 cm ± 0.9 cm. Fish, after freezing, were taken from a different batch than fresh fish. The interval between the fresh fish batches was about 1 week. The weight of one batch of fish from which canned fish was obtained was about 1.5 tons. Fresh (whole) fish was delivered to the fish plant in an iced form and frozen fish in deep-frozen blocks (-22°C, time: 4 months).

### 2.2. Preparation of Canned Food

All canned fishes were obtained under industrial conditions in one of the fishing factory located in Central Europe. The method of canned raw and frozen fish preparation was the same. The blocks of fish delivered to the plant were thawed at 10°C (time 12 h). The fresh and thawed sprats were rinsed. The raw material prepared in this way was immersed in pools of an aqueous solution of salt (20% NaCl, temp. 8°C, and time 20 min). The fish was then smoked, which consisted of two phases: phase I: drying and phase II: smoking (natural smoke from wood chips). The fish was then cooled, and the heads were cut off and packed in oval cans of the Hansa type (148 × 81 × 22 mm). Sunflower oil in the amount of 61-62 g was dosed into cans with smoked fish (the oil originated from a local supplier). The exact weight of the smoked fish and oil in the unit package (can) is shown in Table 1. After addition of the oil, the cans were closed mechanically. Sterilization was carried out at 115°C (flooded autoclave). Sterilization parameters are 15/50/25min (heat-up/specific sterilization/cooling). The smoking and sterilization process parameters used in this study were developed experimentally. The used time and temperature (during smoking and sterilization) are optimal parameters from the point of view of food safety.

### 2.3. Preparation of Samples for Analyses

Ten cans of canned food from each assortment were taken at random for the study. After opening, of the can, the solid parts (fish) were separated from the liquid parts (oil, water). After separating the solid and liquid parts, they were weighed and the percentage in relation to the net weight of the canned food was calculated. Raw fish, smoked fish, and solids parts of canned products (fish) were crushed mechanically using a Zelmer grinder, with a 2 mm mesh screen. The resulting crushed sample was mixed and then analyzed. The oil was also analyzed before sterilization.

### 2.4. Determination of Fat and Water Content

Lipids from raw, smoked fish, and solid and liquid parts of canned fish products were extracted with chloroform and methanol, using the Bligh-Dyer method [12]. The extraction step was performed twice. The lipid content was determined gravimetrically, by evaporating a defined amount of the extract. The water content in raw, smoked fish, and solid parts of canned fish was determined gravimetrically according to AOAC [13] (drying temperature 105°C). The fat content in dry matter (d/m) was determined according to the formula: (fat content/dry matter) × 100%. The dry matter was determined according to the formula: 100% − water content.

### 2.5. Determination of Fatty Acid Profile (EPA and DHA Content)

Fatty acid methyl esters (FAMEs) from lipid extracts were prepared according to AOCS [14] (method Ce 1b-89). FAMEs from sunflower oil (before sterilization) were prepared according to AOCS [14] (method Ce 2-66). The prepared FAMEs were finally extracted with hexane. Next, the FAMEs were separated using a gas chromatography apparatus, coupled with a mass spectrometer (Hewlett Packard G 1800A GCD Series). The conditions of FAMEs separation were carried out in the same way as in the study by Domiszewski [15].

The percentage share of FAs was calculated according to AOCS [14] (method Ce 1b-89).

(1) $$\begin{array}{c} \%\text{fatty acid}=\frac{A_{x}}{A_{T}-A_{\text{IS}}}\times 100, \end{array}$$

where *A*_*X*_ is the area counts of methyl ester, *A*_*T*_ is the total area counts for chromatogram, and *A*_IS_ is the area counts of internal standard (IS).

The absolute EPA and DHA content was calculated according to AOCS [14] (method Ce 1b-89).

(2) $$\begin{array}{c} \text{mg}/\text{g}=\frac{A_{x}\times W_{\text{IS}}\times {\text{CF}}_{x}}{A_{\text{IS}}\times W_{S}\times 1.04}\times 1000, \end{array}$$

where *A*_*x*_ is the area counts of EPA or DHA, *A*_IS_ is the area counts of internal standard, CF_*x*_ is the theoretical detector correction factor for the sample, mg, *W*_*S*_ is the sample weight, mg, and 1.04 is a factor necessary to express result as mg fatty acid/g oil.

As an internal standard, C19 : 0 was used.

EPA and DHA content converted to dry matter (*d*/*w*) was calculated according to the relationship: (fatty acid (g/100 g)/dry matter content) × 100%.

The true retention rate (TR) of DHA and EPA in the solids parts of canned fish (after sterilization) were calculated according to Murphy et al. [9].

(3) $$\begin{array}{c} TR=\frac{\text{EPA and DHA content in fish portion after sterilization }\left(\text{g}\right)\times \text{fish weight after sterilization }\left(\text{g}\right)}{\text{EPA and DHA content in smoked fish portion }\left(\text{g}\right)\times \text{smoked fish weight}\left(\text{g}\right)}\times 100\%, \end{array}$$

where EPA and DHA content in the whole canned fish before sterilization is the EPA and DHA content (g) in a portion of smoked fish that was in the unit package (can) and EPA and DHA content in the whole canned fish after sterilizationis the EPA and DHA content (g) in solid and liquid parts of the unit package.

### 2.6. Determination of Oxidation Level

The quality of fish lipids was determined by analyzing the following factors: peroxide value (PV), p-anisidine value (p-AsV), total oxidation (TOTOX) value, and conjugated dienes (CD) value. PV was determined in the lipid extract by treating with ferric thiocyanate to allow peroxide reduction, as described by Pietrzyk [16]. The red ferric complexes formed were analyzed spectrophotometrically, and the results were expressed as mEqO_2_/kg of lipid. p-AsV was determined in the lipid extract by applying the method of AOCS [14], which is based on the reaction between *α*- and *β*-unsaturated aldehydes (primarily 2-alkenals) and p-anisidine reagent. TOTOX value was calculated using the values determined for peroxide and p-anisidine (2PV + AsV). For the determination of the CD value, the lipid was first extracted following the Bligh and Dyer method. The value was then determined using the AOCS [14] method.

The part of the water layer (WL) in canned food was calculated according to the relationship:

(4) $$\begin{array}{c} WL=\frac{W\;}{m}\times 100\%, \end{array}$$

where *W* is the weight of water layer (g) and *m* is the net weight of packaging (g).

Sodium chloride (salt) content in the solid parts of canned food (fish) was determined by volumetric method according to AOAC [13].

### 2.7. Statistical Analysis

Numbers presented in tables are the mean values of triplicate analyses. The statistical analysis was based on the one-way analysis of variance; homogeneous groups were formed according to the Tukey test for *P* < 0.05. To compare means between two unrelated groups, Student's *t*-test was used. The data were statistically analyzed using STATISTICA (data analysis software system) 2005 version by StatSoft Inc.

## 3. Results and Discussion

### 3.1. Water and Lipid Content and Physical Properties (Share of Solid and Liquid Parts)

Fresh and frozen fish intended for canned food differed in their water and fat content. Fresh sprats were characterized by higher water content (3.1%) and lipid content (6.5%) than frozen sprats (Table 2).

The differences in lipid content resulted from biological factors [17]. Depending on the fishing season, the lipid content of Baltic sprats ranges from 5% to 15.46% [18]. Lower water content in frozen fish may have resulted from the biological factors. The water content in fish decreases with an increase in fat content.

A significant decrease in water content in fish was found after smoking, 7.1%, when the raw material was fresh, and 9.4%, when the raw material was frozen. The decrease in water content in tissue was accompanied by an increase in lipid content (17.9% when the raw material was fresh, and 20.5% when the raw material was frozen). The increase in the content of lipids in wet tissue after fish smoking was caused by dehydration [19]. A decrease in water content occurs in the results of cooking loss, the main component of which is water [20]. Although the fat content in wet weight after sprat smoking increased, some fat losses occurred. This is evidenced by a decrease in the fat content in dry matter (*d*/*m*). After smoking of fresh fish and after freezing storage, the fat content in *d*/*m* decreased by 0.5% and 4.2%, respectively.

After sterilization, a further significant decrease in the water content in the solid parts of canned food (fish) was observed. If smoked (fresh) fish were used for canned food, the water content decreased by 7.3%, and if the raw material was frozen, the decrease was higher and amounted to 8.9%. Also, in this case, the decrease in water content was a result of cooking loss. High temperature during sterilization (115°C) causes protein denaturation. Denaturation of myosin causes *myofibrils* to shrink, and water is excreted [21]. Both thermal stability of myosin and biological factors (fish species and seasonal variations) affect the ability of proteins to hold water [22].

The cooking loss is evidenced by changes in physical parameters of fish and the formation of a water layer (Table 1). After sterilization of canned food, a decrease in the weight of solid parts (fish) by about 8% was found. The decrease in fish weight was accompanied by an increase in the weight of the liquid parts (oil + water) by about 15%. The share of the water layer in canned products obtained from frozen raw material was significantly higher (by 9.5%) compared to canned products obtained from fresh material. The higher share of the water layer in canned products obtained from frozen sprats was probably related to the way the raw material was preserved (i.e., freezing). During frozen storage, the solubility, gelling, and emulsifying capacity of muscle proteins decreases, which affects their ability to bind water [23].

Unfortunately, in canned fish research, the authors do not deal with the cooking loss, and this is a significant problem. In the initial period after sterilization, the weight of fish does not change, but decreases [8]. Some fish plants indicate the net weight of fish after “dripping” on canned food packaging. This is intended to make it easier for consumers to choose the products containing more fish rather than oil. The drop in weight of solid parts in canned food after sterilization was mainly due to cooking loss. Due to the absorption of oil by fish, a better indicator of the size of the cooking loss in canned products is the proportion of the water layer than the change in weight of the fish.

After canned food sterilization, the lipid content of fish increased by about 19% (compared to smoked fish). This observed increase in lipid content was mainly due to diffusion and absorption of oil by fish. A decrease in water content also had some effect, as it causes natural concentration of the other components. It can be concluded from a few papers dealing with this problem that even lipid losses may occur in fish from “oil” canned food after sterilization [24, 25]. It is likely that fat content in fish affects the rate of oil diffusion into the fish.

The presence of acids typical for fish lipids (EPA, DHA) in the oil after sterilization indicates that there were some lipid losses in fish (Table 3). The cooking loss includes not only water but also fat [26]. In this study, the diffusion of oil to sprats caused “distortion” of the lipid content changes. Probably that is why after converting the fat content into dry matter no loss of fat was shown. The scale of oil migration to fish is best demonstrated by an approximately 5-fold increase in the percentage of linoleic acid content in solid parts of canned products (Table 3).

### 3.2. Fatty Acid Profile and EPA and DHA Content

The sprats for canned fish also differed in their fatty acid composition (Table 3).

A higher percentage of EPA and DHA was found in lipids from fresh fish compared to frozen fish. According to Kołakowska et al. [27], the changes in n-3 PUFA in fish during the whole year are the result of interactions between fish development cycle (lipid metabolism) and food (availability, competition).

The smoking of sprats did not significantly affect the percentage share and the EPA and DHA content (g/100 g dry matter) in fish. No significant effect of smoking on the percentage of EPA and DHA in fish lipids was found by Tokarczyk et al. [19]. In turn, Bouzgarrou et al. [28] showed a significant increase in the percentage of these acids in lipids.

After conversion to wet weight, EPA and DHA content in sprats increased significantly by about 20% (Table 4). The observed increase in the content of these acids was mainly due to a decrease in water content. The decrease in water content that occurs during the smoking process may contribute to the increase in the content of some nutrients [29, 30].

The different effects of smoking on the percentage and content of EPA and DHA in fish observed in the literature may depend on the species of fish, different degree of lipids extraction, and smoking parameters [19, 25, 31, 32].

Heat treatments may cause both a decrease in EPA and DHA content in fish [33, 34] and an increase (or no significant effect) [15, 35, 36].

After canned food sterilization, the percentage of EPA and DHA in fish lipids decreased by 22.9%-30.3%. The decrease in the percentage share of EPA and DHA in lipids was caused by two factors. The first one was the increase in fat (oil) content in fish.

This resulted in a natural “dilution,” i.e., a decrease in the percentage of EPA and DHA content in lipids. The second factor was the loss of EPA and DHA caused by cooking loss—this confirms the presence of these acids in the liquid parts of canned food. The available literature shows that EPA and DHA losses in fish after sterilization are between 20% and 40% [10, 11, 37, 38].

Unfortunately, the vast majority of authors presented the results of EPA and DHA analysis in the form of % (this could to some extent distort the “image” of changes). This is confirmed by other authors' research. Replacement of oil with brine in canned fish did not cause a loss of percentage share of EPA and DHA [39].

After converting the results into wet weight, the losses in EPA and DHA content in fish after sterilization were on a level of 9%-17.6% (Table 4). Thus, compared to the results presented as %, the losses were on average 50% lower. Wet weight losses were mainly caused by fat loss, which was confirmed by further analysis of EPA and DHA content in liquid parts and in the whole canned product. This is due to the fact that FAs are part of all lipids, so fat losses automatically cause EPA and DHA losses. Fat losses in fish after heat treatment (accompanied by EPA and DHA losses) were observed, e.g., by Kołakowska et al. [40] and Larsen et al. [41].

This study demonstrated that sterilization resulted in a decrease in the weight of fish (solid parts) in canned food. This further increased the losses of EPA and DHA because lower fish weight is consumed. The calculated true retention rates for EPA and DHA on a level of 70%-77% meant a loss of 30% -23% compared to smoked fish. Therefore, the expression of EPA and DHA content in the form of % or g/100 g does not reflect the actual changes that occurred in acids after sterilization. It is only by taking into account the weight of the fish and the EPA and DHA content of the fish portion that the EPA and DHA losses can be objectively calculated.

After sterilization, TR for EPA and DHA was almost 10% higher in solid parts of canned food obtained from fresh than frozen material. The lower TR found in fish after freezing could be due to a higher loss of lipids (or a higher susceptibility of these acids to temperature). So far, TR has not been calculated in the studies on canned fish. Compared to other heat treatments, it appeared that canned smoked sprats retained more EPA and DHA than, e.g., Pacific saury after deep frying [7].

The minimum daily requirement for EPA and DHA for an adult is 0.25g [42]. Canned smoked sprat was demonstrated to provide a minimum daily dose of EPA and DHA for 5-7 people. Considering a canned product price of 1.5-2 EUR, the cost of a daily dose is between 0.21 and 0.40 EUR.

The conversion of EPA and DHA to dry matter showed that the acid losses in fish were largely equivalent to those calculated on a percentage basis. However, this result may be burdened with some error due to the “absorption” of oil by fish. The lipid content in smoked fish was about 12 g/100 g, so absorption of only 2-3 g of oil causes a significant “distortion” of the result.

Certainly, the oil absorption also affected TR. However, in this case, an absorption of 2-3 g of oil, with the weight of smoked fish about 110 g, has little effect on the final result.

This study demonstrated that the EPA and DHA losses observed in fish after sterilization did not mean their physical destruction. After quantitative determination of EPA and DHA content in the liquid parts of canned food (within the unit pack), it turned out that these acids “passed” from smoked fish to oil. This was confirmed by the analysis of EPA and DHA content in the whole canned food (in the liquid and solid parts). The EPA and DHA content of the whole canned fish was only up to 6% lower than the content of these acids in a portion of fish before sterilization. On the basis of this study, it can be concluded that sterilization does not have a destructive effect on EPA and DHA but only causes the regrouping of acids from fish to oil.

The observed lower TR for EPA and DHA in the solid parts of canned food obtained from frozen/smoked (than fresh) raw material had to be the result of higher fat loss from fish. It can be assumed that changes in the histological structure of the meat and in the functional properties of proteins occurred in the raw material stored in the frozen state [23, 39, 43]. This may have contributed to a higher fat loss in fish during sterilization.

Probably 4%-6% loss of EPA and DHA in the whole canned food resulted from interaction of these compounds with other fish components (mainly proteins). Lipid-protein interactions may occur both during food processing and storage [44]. Some losses of EPA and DHA cannot be excluded increasing the levels of lipid oxidation which occurred after sterilization. However, this hypothesis is contradicted by the analysis of the percentage of linoleic acid content in oil before and after sterilization. The percentage of this acid in the oil after sterilization did not change significantly (although it contains 2 double bonds and is sensitive to oxidation).

Unfortunately, no scientific article on canned fish analyzed EPA and DHA content within the whole canned fish. Therefore, these results could not be compared to other scientific articles.

### 3.3. Lipid Oxidation

Lipid quality is not only determined by the fatty acid content but also the oxidation level. In the extracted lipids from fresh fish, the PV did not exceed 2.9 mEqO_2_/kg of lipid, pAsV 2.4 and CD 0.33% (Table 5).

Oxidation indicators being higher by 55%-116% in sprat after freezing resulted from typical changes that occur in fish during refrigeration storage [45]. Lipoxygenase, which is responsible for lipid peroxidation remains active even at -25 °C [46].

Increased lipid peroxidation indicators were observed after fish smoking. When fresh fish was used for smoking, the increase of primary and secondary oxidation products was 22%-36%. However, if fish after freezing was used, the oxidation level was greater, amounting to 31%-54%. The higher increase in PV, pAsV, and CD observed after smoking in fish that were previously frozen was due to more advanced oxidative changes in lipids. During freezer storage of fish, oxidation products are formed by both enzymatic and free radical pathways. Probably in fresh fish subjected to smoking, lipid oxidation was at the initiation level and in fish after freezing already at the propagation stage.

This explains the faster increase of mainly primary oxidation products in smoked fish that were previously frozen. The factors catalyzing lipid oxidation during smoking include the presence of salt, increased temperature, and forced air circulation (oxygen) [27, 47, 48].

The antioxidant factors occurring during fish smoking mainly include phenolic compounds present in smoke [49]. The available literature shows that fish smoking usually contributes to an increase of primary lipid oxidation products. On the other hand, in the case of secondary oxidation products, smoking can cause both an increase and a decrease in their content. In our opinion, the antioxidant potential of fish muscle tissue and smoking parameters determine whether there is an increase or decrease in secondary oxidation products.

After sterilization of the canned sprat obtained from fresh/smoked raw material, a further increase of PV, p-AsV, and CD by 37%, 28%, and 42%, respectively, was observed (relative to smoked fish). However, after sterilization of the canned sprat obtained from frozen/smoked raw material, the observed increase of p-AsV was only 42%. The two remaining indicators, i.e., PV and CD decreased, respectively, by 37% and 28% (relative to smoked fish).

The generally observed increase of primary and secondary lipid peroxidation products in fish after smoking and sterilization resulted mainly from the effect of high temperature and the presence of salt. It is widely known that fish lipids, due to their high PUFA content, are susceptible to oxidation. One of the prooxidative factors is increased temperature. During fish smoking, the temperature was 60°C (time 30 min) and during sterilization 115°C (time 50 min).

The increased lipid oxidation level observed in the study is compliant with the free radical theory, where primary, followed by secondary, oxidation products are formed [50]. This process was particularly pronounced after the sterilization of canned fish obtained from frozen material. In solid parts of the canned fish (fish meat), a decrease of the content of primary oxidation products was observed (PV, CD) which was accompanied by the increase of secondary products (p-AsV). Peroxides and hydroperoxides are highly unstable compounds and their decompositions increased in higher temperature [50]. According to Aubourg [51], during heating, the increase of peroxides concerns fish muscle tissue with lower oxidation level, whereas with higher levels of 8–12 mEq O_2_/kg of lipids their decomposition takes place. In general, CD was characterized by a greater increase after smoking and sterilization. This was a result of the fact that conjugated dienes are formed at the earliest lipid oxidation stage [52].

The increase of oxidation level in fish after can sterilization was observed by Morales et al. [53] and Medina et al. [54], and a decrease by Aubourg et al. [55]. To our opinion, such a varied impact of sterilization on the level of lipid peroxidation in canned fish depends largely on its level before sterilization.

Certainly, salt also had an effect on lipid peroxidation in fish after heat treatment (smoking, sterilization). In order to provide fish with suitable sensory properties in cans, they are subject to brining. Depending on the batch of canned fish, the NaCl content in fish meat was 2.54 g-2.76 g/100 g (Table 5). Salt is known for its prooxidative properties [56].

Apart from the prooxidative factors that occur during canned meat production (temperature, salt), antioxidative factors can also be observed. Fish intended for cans were subject to smoking that is saturated with smoke. The composition of smoke includes numerous compounds that exhibit strong antioxidative properties (mainly phenols) [57]. The limitation of oxidation could be to some extent produced by the amino acids, nucleotides, peptides, and Maillard reaction products present in fish [58, 59]. Proteins and peptides, due to their capacity to scavenge free radicals and to chelate metals, are believed to be important antioxidants present in meat [60].

Presently, no legal regulations are in place in terms of the maximum lipid peroxidation level in fish (regulations only concern nutraceuticals and dietary supplements). Some associations recommend that PV should not exceed 5-10 mEq O_2_/kg lipids and p-AsV 20-30 [61, 62].

The technological process also resulted in a significant increase of the analyzed oxidation indicators (by 24% -54%) in liquid parts of the can contents (oil). The higher oil oxidation level was observed in cans obtained from frozen than from fresh material.

It appears that temperature and cooking loss had major impacts on oil oxidation during sterilization. Prior to sterilization, sunflower oil had low oxidation indicators. As a result of lipid leaking (with higher oxidation level) from fish, they were mixed with oil, which resulted in increased oxidation. Perhaps this was the reason why the oil from cans obtained from frozen fish was characterized by higher oxidation levels. Certainly, with the heat leakage, a certain portion of antioxidative compounds, such as phenols and peptides penetrated to the liquid ingredients of cans. For this reason, the heat leakage could also restrict the increase of oil oxidation to some degree.

The mutual exchange of lipids between oil and fish was also observed by Aubourg et al. [55] and Tarley et al. [63]. The study conducted by Medina et al. [64] showed that antioxidants play a significant role in the restriction of lipid oxidation in the liquid contents of cans. Medina et al. [64] determined the lowest lipid oxidation level in cans with extra virgin olive oil (that is rich in natural antioxidants).

Perhaps, the low oxygen level in the cans had some impact on the lipid peroxidation level in the canned fish (there is a very small space between the contents of the can and the lid). The study conducted by Marciniak–Łukasiak et al. [65] demonstrated that oxygen dissolved in oil is not as important oxidation factor as the oxygen contained in the packaging, above the oil surface.

The present study demonstrated that not only fish but also oil used in the cans was characterized by good quality lipids. The determined oxidation factors did not exceed the limits set for vegetable oils PV < 10 mEq O_2_/kg lipids [66]. None of the investigated oils had PV in excess 3.5, p-AsV 2.7 and index TOTOX 26.

## 4. Conclusions

Unequivocal statement of the effect of the technological process on EPA and DHA content in canned smoked sprat in oil is problematic. Considering the whole canned food (fish and oil), EPA and DHA losses after sterilization were small and did not exceed 6%. Since only fish is consumed from canned food in oil, it retains 70-77% of EPA and DHA compared to smoked fish (before sterilization). The remaining part of the acids from the fish is transferred to oil together with the fat. After sterilization, fresh/smoked fish retain almost 10% more EPA and DHA than fish after freezing. During the production of canned food, EPA and DHA are mainly regrouped (eluted) from fish to oil. The expression of EPA and DHA content in the form of % or g/100 g does not reflect the actual changes in acids after sterilization. Despite the double dose of heat that occurred during the canned sprat production process (smoking, sterilization) PV in fish and in oil did not exceed 10 (mEqO_2_/kg of lipid) and p-AsV did not exceed 20. This means that these lipids were characterized by good quality. Due to the absorption of oil by fish, a better indicator of changes in the physical parameters of canned fish after sterilization is the analysis of the proportion of the water layer rather than mass measurement.

## Figures and Tables

**Table 1:** The weight (g) of the smoked fish, oil, and solid and liquid parts from canned fish in the unit package.

Raw material		Canned food		
S	O	Sp	Lp	WL
Raw material: fresh/smoked fish				
110.0^d^ ± 1.0 (64%^A^)	62.0^a^ ± 0.6 (36%^A^)	101.0^c^ ± 1.1 (58.7%^A^)	71.0^b^ ± 0.6 (41.3%^A^)	5.81^A^ ± 0.20
Raw material: frozen/smoked fish				
112.0^d^ ± 1.0 (64.7%^A^)	61.0^a^ ± 0.8 (35.3%^A^)	103.0^c^ ± 1.2 (59.5%^A^)	70.0^b^ ± 0.7 (40.5%^A^)	6.36^B^ ± 0.25

(percentage in parentheses) S: smoked fish; O: sunflower oil; Sp: solid parts from canned product (fish after sterilization); Lp: liquid parts from canned product (oil + water after sterilization); WL: water layer (%); ^a, b, c^values represented by the same letters in row are not significantly different from each other with *P* ≤ 0.05. ^A, B^Values represented by the same letters in column are not significantly different from each other with *P* ≤ 0.05.

**Table 2:** Water and lipid content in raw fish, smoked fish, and solid and liquid parts of canned fish.

	Water content g/100 g	Lipid content g/100 g (w/m)	Lipid content g/100 g (d/m)
Raw material: fresh/smoked fish			
R	75.54^d^ ± 0.38	10.03^a^ ± 0.20	41.01^a^ ± 0.98
S	70.18^c^ ± 0.49	11.83^b^ ± 0.27	39.67^a^ ± 1.11
Sp	65.03^b^ ± 0.39	13.98^c^ ± 0.27	39.98^a^±1.00
Lp	14.08^a^ ± 0.10	85.92^d^ ± 1.89	
Raw material: frozen/smoked fish			
R	73.28^d^ ± 0.37	9.42^a^ ± 0.15	35.28^a^ ± 0.81
S	66.42^c^ ± 0.30	11.35^b^ ± 0.24	33.80^a^ ± 0.95
Sp	60.51^b^ ± 0.27	13.54^c^ ± 0.31	34.06^a^ ± 0.99
Lp	15.71^a^ ± 0.10	84.29^d^ ± 1.50	

*w*/*m*: wet matter; *d*/*w*: dry matter; R: raw fish; S: smoked fish; Sp: solid parts from canned product (fish) (after sterilization); Lp: liquid parts from canned product (oil + water) (after sterilization); ^a, b, c^values represented by the same letters in column (for the same parameter: fresh/frozen fish) are not significantly different from each other with *P* ≤ 0.05.

**Table 3:** Composition of fatty acids (%) in the raw materials (raw fish, smoked fish, and oil) and solid and liquid parts of canned smoked sprat.

	Raw material: fresh/smoked fish					Raw material: frozen/smoked fish				
	R	S	O	Sp	Lp	R	S	O	Sp	Lp
C14:0	5.48^d^	5.38^d^	0.03^a^	4.11^c^	0.20^b^	6.28^d^	6.41^d^	0.06^a^	4.63^c^	0.33^b^
C15:0	1.09^c^	1.07^c^	ND	0.86^b^	0.03^a^	1.37^c^	1.41^c^	ND	1.05^b^	0.05^a^
C16:0	23.41^d^	22.94^d^	5.63^a^	19.11^c^	6.03^b^	22.51^c^	22.49^c^	6.36^a^	17.61^b^	6.92^a^
C 17 : 0	0.09^ab^	0.10^b^	TA	0.08^a^	TA	0.22^b^	0.22^b^	TA	0.17^a^	TA
C16 : 1	5.51^d^	5.58^d^	0.25^a^	4.62^c^	0.39^b^	5.49^d^	5.61^d^	0.10^a^	4.02^c^	0.34^b^
C17 : 1	1.35^c^	1.36^c^	TA	1.08^b^	0.04^a^	0.92^c^	0.89^c^	TA	0.63^b^	0.04^a^
C18 : 0	2.58^a^	2.47^a^	4.92^c^	3.09^b^	4.78^c^	2.65^a^	2.61^a^	4.27^c^	3.21^b^	4.09^c^
C18 : 1	26.61^a^	26.67^a^	25.32^a^	26.16^a^	25.06^a^	29.54^c^	29.18^c^	24.99^a^	27.77^b^	25.35^a^
C18 : 2 n-6	3.01^a^	3.12^a^	62.89^c^	16.50^b^	61.72^c^	4.67^a^	4.73^a^	63.55^c^	22.05^b^	61.21^c^
C20 : 0	0.34^b^	0.35^b^	0.30^a^	0.32^a^	0.31^a^	0.23^b^	0.23^b^	0.15^a^	0.21^b^	0.15^a^
C18 : 3 n-3	2.68^c^	2.67^c^	TA	2.03^b^	0.08^a^	2.35^c^	2.36^c^	TA	1.66^b^	0.10^a^
C20 : 1	1.33^d^	1.39^d^	0.15^a^	1.09^c^	0.21^b^	0.86^c^	0.86^c^	0.25^a^	0.69^b^	0.27^a^
C18 : 4 n-6	2.62^c^	2.61^c^	ND	2.00^b^	0.07^a^	1.48^c^	1.51^c^	ND	1.13^b^	0.05^a^
C20 : 2 n-6	0.35^b^	0.37^c^	ND	0.31^a^	TA	0.46^c^	0.48^c^	ND	0.36^b^	0.02^a^
C22 : 0	ND	ND	0.51^b^	0.12^a^	0.48b	ND	ND	0.27^b^	0.09^a^	0.25^b^
C22 : 1	0.85^c^	0.84^c^	TA	0.66^b^	0.02^a^	0.74^c^	0.73^c^	TA	0.52^b^	0.03^a^
C20 : 4 n-6	0.40^c^	0.40^c^	ND	0.33^b^	0.01^a^	0.42^c^	0.41^c^	ND	0.28^b^	0.02^a^
C20 : 4 n-3	0.29^b^	0.29^b^	ND	0.24^a^	TA	0.35^c^	0.36^c^	ND	0.27^b^	0.01^a^
EPA n-3	8.03^c^	8.01^c^	ND	6.25^b^	0.21^a^	5.48^c^	5.46^c^	ND	3.82^b^	0.22^a^
C24 : 1	0.46^c^	0.45^c^	ND	0.36^b^	0.01^a^	1.78^c^	1.77^c^	ND	1.27^b^	0.07^a^
C22 : 5 n-3	0.31^b^	0.31^b^	ND	0.25^a^	TA	0.56^c^	0.57c	ND	0.41^b^	0.02^a^
DHA n-3	13.21^c^	13.62^c^	ND	10.43^b^	0.35^a^	11.64c	11.71^c^	ND	8.15^b^	0.46^a^
SFA	32.99^c^	32.31^c^	11.39^a^	27.69^b^	11.83^a^	33.26^d^	33.37^d^	11.11^a^	26.97^c^	11.79^b^
MUFA	36.11^c^	36.29^c^	25.72^a^	33.97^b^	25.73^a^	39.33^c^	39.04^c^	25.34^a^	34.90^b^	26.10^a^
PUFA	30.90^a^	31.40^a^	62.89^c^	38.34^b^	62.44^c^	27.41^a^	27.59^a^	63.55^c^	38.13^b^	62.11^c^
n-6	6.38^a^	6.50^a^	62.89^c^	19.14^b^	61.80^c^	7.03^a^	7.13^a^	63.55^c^	23.82^b^	61.30^c^
n-3	24.52^c^	24.90^c^	—	19.20^b^	0.64^a^	20.38^c^	20.46^c^	—	14.31^b^	0.81^a^
n-6/n-3	0.26^a^	0.26^a^	—	1.00^b^	96.56^c^	0.34^a^	0.35^a^	—	1.66^b^	75.68^c^

R: raw fish; S: smoked fish; O: sunflower oil; Sp: solid parts from canned product (fish); Lp: liquid parts from canned product (oil + water after sterilization); ND: nondetectable; TA: trace amounts; ^a, b, c^values represented by the same letters in row (for the same parameter: fresh/frozen fish) are not significantly different from each other with *P* ≤ 0.05.

**Table 4:** Contents of the sum of EPA and DHA, TR in raw fish, smoked fish, and solid and liquid parts of canned fish and whole canned products.

	%	g/100 g wet matter	g/100 g dry matter	TR	Whole canned products	
					B/s	A/s
Raw material: fresh/smoked fish						
R	21.24^c^ ± 0.85	1.77^b^ ± 0.08	7.24^c^ ± 0.39		2.33^A^	2.24^A^
S	21.63^c^ ± 0.97	2.12^d^ ± 0.10	7.14^c^ ± 0.38			
Sp	16.68^b^ ± 0.62	1.93^c^ ± 0.08	5.52^b^ ± 0.26	76.8^b^		
Lp	0.56^a^ ± 0.03	0.40^a^ ± 0.02	0.47^a^ ± 0.03			
Raw material: frozen/smoked fish						
R	17.12^c^ ± 0.77	1.37^b^ ± 0.06	5.13^c^ ± 0.26		1.84^B^	1.74^A^
S	17.17^c^ ± 0.67	1.65^c^ ± 0.07	4.90^c^ ± 0.23			
Sp	11.97^b^ ± 0.62	1.36^b^ ± 0.07	3.44^b^ ± 0.19	70.0^a^		
Lp	0.68^a^ ± 0.03	0.49^a^ ± 0.02	0.58^a^ ± 0.03			

TR: true retention rate; B/s: whole canned product before sterilization; A/s: whole canned product after sterilization; R: raw fish; S: smoked fish; Sp: solid parts from canned product (fish) (after sterilization); Lp: liquid parts from canned product (oil + water) (after sterilization); ^a, b, c^values represented by the same letters in column (for the same parameter: fresh/frozen fish) are not significantly different from each other with *P* ≤ 0.05. ^A, B^Values represented by the same letters in row are not significantly different from each other with *P* ≤ 0.05.

**Table 5:** Oxidation level (in the extracted lipids from oil, raw fish, smoked fish, and solid and liquid parts of canned fish) and salt content in solid parts of canned sprat.

	PV (mEqO_2_/kg)	p-AsV	TOTOX	CD (%)	NaCl (g/100 g)
Raw material: fresh/smoked fish					
O	2.09^a^ ± 0.11	2.03^a^ ± 0.12	6.21^a^ ± 0.26	0.24^a^ ± 0.02
R	2.88^b^ ± 0.12	2.31^ab^ ± 0.11	8.07^b^ ± 0.29		
S	3.72^c^ ± 0.22	2.82^c^ ± 0.15	10.25^c^ ± 0.43	0.45^c^ ± 0.03	
Sp	5.09^d^ ± 0.29	3.61^d^ ± 0.21	13.79^d^ ± 0.60	0.64^d^ ± 0.04	2.54 ± 0.08
Lp	2.76^b^ ± 0.20	2.52^bc^ ± 0.13	8.03^b^ ± 0.30	0.31^b^ ± 0.02	
Raw material: frozen/smoked fish				
O	1.95^a^ ± 0.10	1.99^a^ ± 0.10	5.89^a^ ± 0.25	0.26^a^ ± 0.02	
R	6.21^c^ ± 0.29	4.16^c^ ± 0.18	16.58^c^ ± 0.63	0.51^c^ ± 0.03	
S	8.69^d^ ± 0.48	5.45^d^ ± 0.28	22.84^e^ ± 0.92	0.79^d^ ± 0.05	
Sp	5.62^c^ ± 0.30	7.92^e^ ± 0.43	19.16^d^ ± 0.77	0.37^b^ ± 0.02	2.76 ± 0.09
Lp	3.01^b^ ± 0.2	2.69^b^ ± 0.12	8.71^b^ ± 0.34	0.34^b^ ± 0.02	

O: sunflower oil; R: raw fish,; S: smoked fish; Sp: solid parts from canned product (fish) (after sterilization); Lp: liquid parts from canned product (oil + water) (after sterilization); ^a, b, c^values represented by the same letters in column (for the same parameter: fresh/frozen fish) are not significantly different from each other with *P* ≤ 0.05.

## Data Availability

The data used to support the findings of this study are included within the article.
